# Tumor-associated neutrophils in breast cancer: an angel or a devil?

**DOI:** 10.3389/fimmu.2025.1593156

**Published:** 2025-06-11

**Authors:** Siyuan Wen, Tianli Feng, Yu Fan

**Affiliations:** ^1^ Faculty of Clinical Medicine, Southwest Medical University, Luzhou, China; ^2^ Department of Oncology, The Affiliated Hospital of Southwest Medical University, Luzhou, China

**Keywords:** breast cancer, tumor-associated neutrophils, immune cell, immune microenvironment, targeted therapy

## Abstract

Breast cancer is the most common malignant tumor in women, ranking first globally in both incidence and mortality rates among female malignancies, posing a severe threat to women’s physical and mental health. Neutrophils are recognized as the first line of host defense against pathogens and exert beneficial effects in the body. However, emerging evidence has demonstrated that tumor-associated neutrophils (TANs) exhibit a dual role in breast cancer progression and prognosis. Therefore, elucidating their molecular mechanisms may provide novel insights for targeted therapies, potentially improving clinical outcomes for breast cancer patients. This review summarizes the interplay between TANs and breast cancer, their underlying mechanisms, and their potential as immunotherapeutic targets.

## 1 Introduction

Breast cancer, a highly prevalent malignancy in women, exhibits a growing global disease burden. According to the 2020 Global Cancer Statistics by the International Agency for Research on Cancer (IARC), breast cancer has surpassed lung cancer as the most common malignancy among women in 185 countries, with an age-standardized incidence rate of 47.8 per 100,000 (1). Tumor biology studies highlight dynamic interactions between the tumor microenvironment (TME) and cancer progression, mediated by complex molecular regulatory networks (2). Deciphering key TME components and their mechanisms has thus emerged as a critical research focus in oncology. Neutrophils, as core effector cells in the TME, have gained significant attention. Clinical pathological evidence indicates that there are significant differences in the levels and clinical significance of neutrophil infiltration among different breast cancer subtypes, with these recruited granulocytes termed tumor-associated neutrophils(TANs) (3). Triple-negative breast cancer (TNBC) exhibits the highest TANs positivity rate (88%), which may be closely related to the activation of pro-inflammatory factors in its tumor microenvironment and increased circulating neutrophil counts (3). The HER2-positive (HER2+) subtype has the second-highest TANs density (positivity rate 53%) and is associated with more aggressive clinical features, including larger tumor size, higher histological grade, and elevated lymph node metastasis rates (4). In Luminal B-type patients, TANs infiltration is significantly correlated with tumor progression markers such as lymphovascular invasion, high proliferative index (Ki67 ≥13.25%), and regional lymph node metastasis, which may be linked to its higher histological grade and low hormone receptor expression (5). In contrast, Luminal A-type tumors show the lowest TANs infiltration (positivity rate only 5%) due to high hormone receptor (ER/PR) expression, low proliferative activity, and a less pro-inflammatory microenvironment. This subtype is associated with favorable prognostic indicators such as low lymph node metastasis rates and smaller tumor size (3). Traditionally viewed as innate immune defenders that suppress tumors via phagocytosis and reactive oxygen species (ROS) release, recent studies reveal TANs’ functional heterogeneity and phenotypic plasticity, enabling dual roles in both anti-tumor immunity and pro-tumor progression (6). Notably, although the 5-year survival rate for breast cancer patients can reach 90%, treatment-induced physiological dysfunction and psychological disorders severely reduce patients’ quality of life (7, 8). Therefore, investigating the mechanisms of TANs in breast cancer progression and developing targeted immunotherapeutic strategies based on TANs phenotype regulation hold significant clinical value for achieving precision medicine and improving patient prognosis. This review systematically examines the regulatory mechanisms of TANs in breast cancer initiation, progression, metastasis, and therapy resistance, focusing on three key scientific questions (1): interactions between TANs, tumor cells, and immune cells (2); molecular networks governing TAN phenotypic switching (3); translational potential of TANs as diagnostic biomarkers and therapeutic targets. By integrating recent advances, this work aims to provide a theoretical foundation for understanding the breast cancer immune microenvironment and to guide the development of novel therapeutic strategies.

## 2 Release and recruitment of TANs

**Figure 1:**
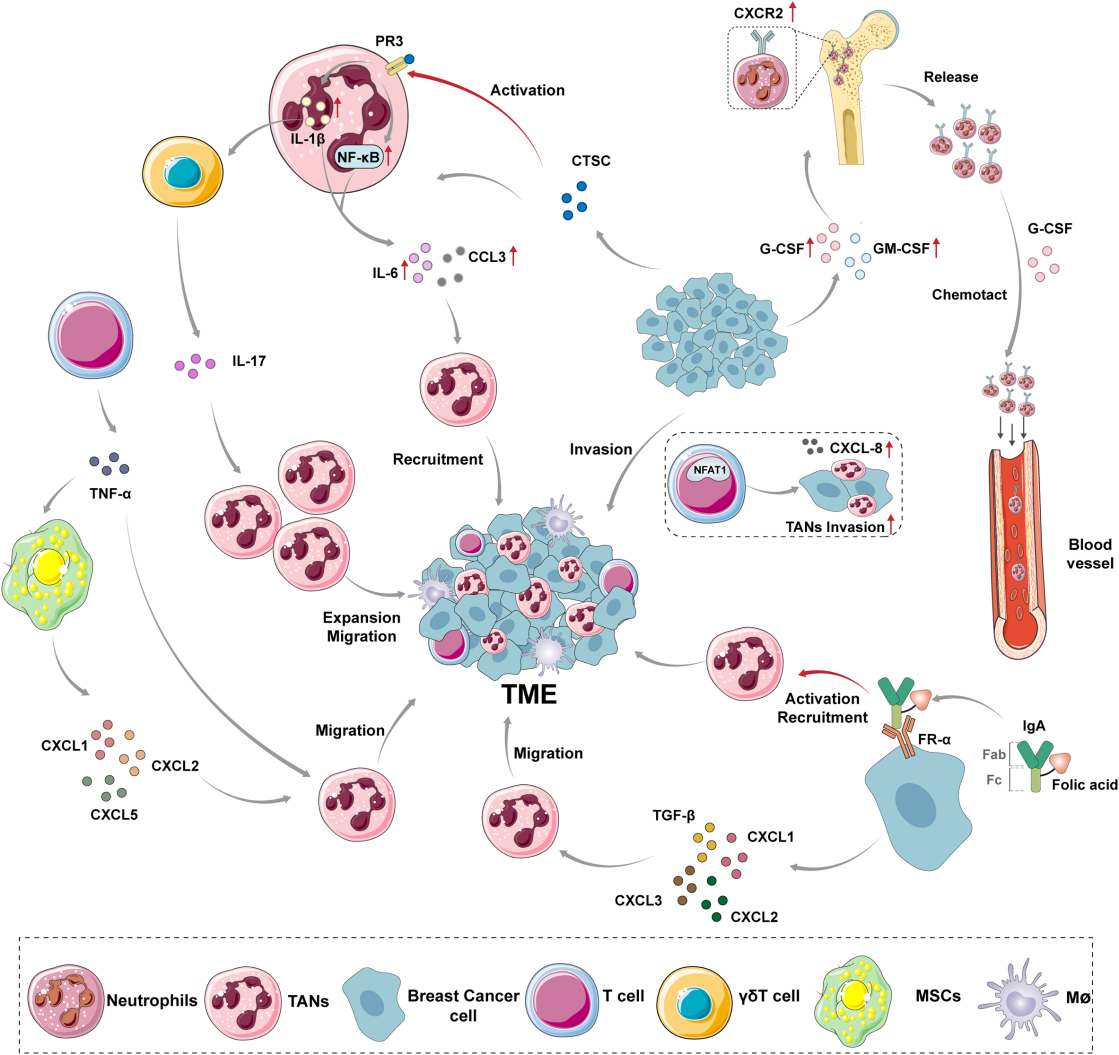
The mechanism of TANs recruitment to breast cancer TME. Under normal conditions, the expression of CXCR2 is up-regulated on the surface of mature neutrophils, which are released from the bone marrow into the bloodstream under the chemotactic effect of G-CSF. However, the recruitment of TANs is regulated by different mediators secreted by various cells and some nutrient factors *in vivo*. G-CSF and GM-CSF provide a source of TANs recruitment by promoting the release of neutrophils from bone marrow. NFAT1 promotes the infiltration of neutrophils in tumors by promoting the expression of CXCL-8 in breast cancer cells. CTSC secreted by breast cancer cells promotes the processing of IL-1β and the activation of NF-κB by activating PR3 on the neutrophil membrane, resulting in the enhanced expression of IL-6 and CCL3, and recruits neutrophils to the tumor site. The activated IL-1β can also induce γδT cells to express IL-17, promoting the amplification and migration of neutrophils. The IgA Fc-folate coupling formed by the Fc fragment of IgA molecules and folate molecules binds to FR-α on TNBC cells, activating and recruiting neutrophils. CXCL1, CXCL2, CXCL3 and TGF-β secreted by TNBC cells can synergistically induce neutrophil migration. TNF-α secreted by T cells can not only promote the recruitment of neutrophils by itself, but also cause MSCs secreting CXCL1, CXCL2 and CXCL5 to promote the migration of neutrophils to breast cancer TME. CXCR2, CXC receptor 2; TME, tumor microenvironment; TANs, tumor-associated neutrophils; MSCs, mesenchymal stromal cells; G-CSF, Granulocyte Colony Stimulating Factor; GM-CSF, Granulocyte-macrophage Colony Stimulating Factor; NFAT1, Nuclear Factor of Activated T-cells 1; PR3, Protease 3; CTSC, tumor-secreted protease cathepsin C; NF-κB, nuclear factor-kB; IL-1β, Interleukin-1β; IL-6, Interleukin-6; CCL3, CC Motif Chemokine Ligand-3; IL-17, Interleukin-17; FR-α, Folate Receptor Alpha; TGF-β, Transforming Growth Factor Beta; TNF-α,Tumour Necrosis Factor Alpha; CXCL1, CXC Motif Chemokine Ligand-1; CXCL2,CXC Motif Chemokine Ligand-2; CXCL3, CXC Motif Chemokine Ligand-3; CXCL5,CXC Motif Chemokine Ligand-5.

Neutrophils, as the predominant myeloid-derived granulocyte subpopulation, constitute 50%-70% of peripheral blood leukocytes. Their production, mobilization, and migration are dynamically regulated by the balance between the CXC receptor 4 (CXCR4)-CXC Motif Chemokine Ligand-12 (CXCL12) axis and CXC receptor 2 (CXCR2)–CXC Motif Chemokine Ligand-1/2 (CXCL1/2) signaling pathways (9, 10). Mature neutrophils detach from bone marrow stroma through synergistic interactions between surface CXCR2 and granulocyte colony-stimulating factor (G-CSF), subsequently entering the TME via peripheral circulation. G-CSF promotes neutrophil egress by downregulating CXCR4 expression, while CXCR2 ligands (e.g., CXCL1/2/3/5/8) establish chemotactic gradients guiding directional infiltration (11). Notably, nuclear factor of activated T cells 1 (NFAT1) in breast cancer cells transcriptionally upregulates CXC chemokine ligand 8 (CXCL8/IL-8) expression, driving localized neutrophil accumulation within tumors (12). Recent studies reveal that breast cancer-derived cathepsin C (CTSC) activates neutrophil membrane protease 3 (PR3), triggering Interleukin-1β (IL-1β)/nuclear factor-kB (NF-κB) signaling cascades through pro-IL-1β cleavage. This process induces Interleukin-6 (IL-6) and CC Motif Chemokine Ligand-3 (CCL3) paracrine secretion, forming an autocrine-paracrine positive feedback loop that amplifies sustained neutrophil recruitment (13, 14). Furthermore, IL-1β upregulates CXCR2 ligand expression via γδT cell-dependent Interleukin-17 (IL-17) pathways, enhancing neutrophil expansion and migration, highlighting functional crosstalk among immune cell subsets through cytokine networks (15).In TNBC, neutrophil recruitment exhibits marked heterogeneity. Clinical cohorts demonstrate folate receptor-α (FR-α) overexpression in 35%-80% of TNBC patients, independently correlating with shortened progression-free survival (16, 17). Mechanistically, IgA Fc-folate conjugates targeting FR-α elicit FcαR-mediated neutrophil degranulation and antibody-dependent cellular cytotoxicity (ADCC), offering novel immunotherapeutic strategies. Concurrently, TNBC-secreted CXCR2 ligands (CXCL1/2/3) synergize with TGF-β: TGF-β upregulates CXCR2 expression through Smad3-dependent pathways, while CXCL1/2/3 enhance neutrophil migratory activity via PI3K/Akt signaling. Their combined effects correlate positively with tumor histologic grade and metastatic potential (18). Notably, T cell-derived TNF-α directly activates NF-κB in neutrophils and induces mesenchymal stromal cells (MSCs) to secrete CXCL1/2/5, creating cascade amplification (19, 20). Recent studies in lung adenocarcinoma models have revealed that extramedullary hematopoietic organs such as the spleen can serve as secondary reservoirs for neutrophils, which participate in TME infiltration through CXCR2 ligand-dependent mechanisms (21). Although this phenomenon has not been definitively validated in breast cancer, these findings highlight the need to systematically elucidate the heterogeneous origins of neutrophils and their spatiotemporal regulatory networks. Such insights could provide a theoretical foundation for developing targeted intervention strategies against tissue-specific microenvironments. This article summarizes the currently known mechanisms by which TANs enter the breast cancer TME (**Figure 1**).

## 3 Plasticity and diversity of TANs

The plasticity and functional diversity of neutrophils constitute the biological basis for the dual regulatory potential of TANs, enabling them to undergo phenotypic polarization in response to heterogeneous signaling stimuli within the TME (22, 23). Recent single-cell multi-omics studies have overturned the classical N1/N2 dichotomy model: Ng et al. (24) systematically revealed the high heterogeneity of TANs for the first time by integrating single-cell RNA sequencing (scRNA-seq) and assay for transposase-accessible chromatin sequencing (ATAC-seq) data of multi-organ neutrophils in an orthotopic pancreatic cancer mouse model, defining three functionally distinct subsets—T1, T2, and T3 neutrophils. Notably, regardless of their initial maturation status, neutrophils entering the breast cancer microenvironment are reprogrammed into the terminal T3 subset. This phenomenon is highly conserved in pan-cancer analyses (including breast cancer), suggesting that the T3 subset may represent a key effector cell population driving tumor promotion across cancer types. Additionally, Dai et al (25). utilized scRNA-seq technology to identify three functionally heterogeneous subpopulations of TANs in breast cancer: Neutrophil_IFIT1, Neutrophil_DUSP6, and Neutrophil_S100A1.The IFIT1^+^ subpopulation exhibits high expression of ISG15, MX1, and IFIT1, demonstrating antigen-presenting capabilities. It enhances the efficacy of anti-PD-1 therapy by activating CD8^+^ T cells. However, its immune-activating effects can be counteracted by Programmed cell death 1 ligand 1^+^ (PD-L1^+^) TANs via the JAK-STAT3 pathway. Furthermore, the absence of DHX9-STAT1 co-regulation significantly suppresses its ISG15/IFIT1 expression (26). The DUSP6^+^subpopulation shows reduced chemotaxis and phagocytic capacity after intraoperative radiotherapy (IORT). Its kinase network activation state is closely linked to breast cancer hormone receptor (HR) and HER2 status. Metabolically, it relies on the pyruvate carboxylase pathway and contributes to the formation of an immunosuppressive microenvironment through metabolic reprogramming (27). The S100A1^+^subpopulation promotes a pro-inflammatory microenvironment and accelerates angiogenesis and matrix remodeling by releasing leukotriene B4(LTB4), ROS, and matrix metalloproteinase-9(MMP9), directly facilitating breast cancer metastasis. Its metabolic adaptation is characterized by high expression of glycolysis- and oxidative stress-related genes, enabling it to sustain pro-tumor functions in hypoxic microenvironments (27). Additionally, it promotes lymph node metastasis through the NECTIN2-TIGIT-mediated immune escape mechanism (28). The heterogeneity of neutrophils in breast cancer is manifested not only in functional differentiation but also in physical density and developmental stages. Neutrophils in breast cancer patients are classified into low-density neutrophils (LDNs) and high-density neutrophils (HDNs) based on density. LDNs comprise a mixed population of immature (banded nuclei) and mature (segmented nuclei) neutrophils that suppress T-cell proliferation by releasing arginase1 (ARG1) and ROS, thereby promoting breast cancer immune escape (15, 29–31). Immature LDNs (iLDNs) highly express liver-homing receptors (e.g., CXCR4) and drive breast cancer liver metastasis by forming pre-metastatic niches, which are significantly associated with reduced overall survival in patients (15). Under physiological conditions, HDNs account for 95% of circulating neutrophils and exert anti-infective functions through phagocytosis and neutrophil extracellular traps (NETs) release. In the breast cancer microenvironment, tumor-derived Transforming Growth Factor Beta (TGF-β) induces HDN-to-LDN conversion via the SMAD3 signaling pathway, while spontaneous HDN conversion in advanced-stage patients may be linked to mitochondrial dysfunction (31, 32). Additionally, Sagiv et al. (32) identified the presence of myeloid-derived suppressor cells (MDSCs) in the peripheral blood of 4T1 murine breast cancer models. TANs and MDSCs exhibit significant associations. Granulocytic MDSCs (G-MDSCs) and pro-tumoral TANs overlap phenotypically and functionally, both suppressing T cell activity through mechanisms such as ARG1 and ROS (33). MDSCs also secrete matrix metalloproteinases (MMPs) to promote metastasis, while TANs-secreted MMP9 further amplifies this process, creating a vicious cycle (24). Importantly, MDSCs possess plasticity to differentiate into tumor-associated macrophages (TAMs) or TANs (34). TAMs also play critical roles in tumorigenesis and progression. IL-6 produced by TANs upregulates PD-L1 through STAT3 pathway activation, synergizing with immune checkpoint molecules such as PD-L1 and Tim-3 expressed by TAMs to further suppress T cell activity (35). Polymorphonuclear MDSCs (PMN-MDSCs) share CD11b^+^/CD14^-^/CD15^+^ markers with neutrophils but exhibit elevated expression of immunosuppressive molecules such as PD-L1 and inducible nitric oxide synthase (iNOS) (36). In both 4T1 murine models and breast cancer patients, circulating PMN-MDSC levels positively correlate with tumor burden, metastatic burden, and immune checkpoint molecule expression (e.g., CTLA-4), highlighting their potential as prognostic biomarkers (37). Importantly, PMN-MDSCs and TANs exhibit partial phenotypic and functional overlap, necessitating further discrimination via single-cell transcriptomics or surface markers (e.g., LOX-1) (38, 39).

In summary, current studies indicate that the nomenclature and classification of TANs exhibit significant complexity. Depending on different functions or experimental models, neutrophils are assigned various names (e.g., PMN-MDSCs, LDN), but these terms likely reflect their functional plasticity rather than distinct subsets. The lack of standardized surface marker combinations limits the comparability of results across studies. Furthermore, TANs functions are highly dependent on the TME. While *in vitro* polarization models can partially simulate TANs phenotypes, they fail to fully replicate the dynamic cellular interactions within the TME. For instance, co-culture systems with T cells cannot adequately reflect the regulatory effects of macrophages, NK cells, or other immune components on TANs *in vivo*. Future research should integrate single-cell multi-omics technologies to establish unified molecular classification criteria for TANs. Additionally, developing organoid models that mimic the TME’s intricate interactions will be critical for advancing our understanding of TAN roles in tumor progression and therapy.

## 4 Apoptosis of TANs

The dynamic balance between the generation and apoptosis of neutrophils is a core mechanism for maintaining immune homeostasis in the body. Under physiological conditions, after being released from the bone marrow into peripheral blood, neutrophils exhibit a short-lived survival characteristic with a half-life of approximately 19 hours. Upon migrating into tissues, they can survive for 1–3 days before being cleared by macrophages via programmed apoptosis (40, 41). This apoptotic process is precisely regulated by intrinsic and extrinsic signaling pathways: the intrinsic pathway involves mitochondrial membrane potential collapse-mediated cytochrome c release, which forms the apoptosome complex with Apaf-1, ATP/dATP, and procaspase-9, subsequently activating downstream caspase-3 and initiating a cascade reaction (42–44). The extrinsic pathway is triggered by death receptors (Fas/TNFR1) or intracellular stress factors (ROS/cathepsin D/G), executing apoptosis through the caspase-8/3 signaling axis via cleavage of aspartate residue-containing substrate proteins (45, 46). Notably, the breast cancer microenvironment induces significant biological reprogramming of TANs. Emerging evidence reveals that TANs infiltrating the tumor stroma can be “educated” into long-lived pro-tumor subsets (survival extended to 135 hours) within 24 hours (24), with their survival duration significantly surpassing that of neutrophils under physiological conditions (p < 0.001). This provides a temporal window for sustained interactions between TANs and tumor cells. The key scientific questions currently lie in (1): whether TANs achieve prolonged survival through apoptotic regulatory pathways (e.g., mitochondrial-dependent apoptosis resistance, death receptor signaling inhibition, or overexpression of anti-apoptotic proteins); and (2) whether such apoptotic imbalance leads to tumor immune editing dysregulation and malignant progression. Systematic elucidation of the mechanisms underlying TANs’ apoptotic evasion and their relationship with breast cancer progression will help uncover novel therapeutic targets in the tumor immune microenvironment, constituting critical unresolved scientific issues for future research.

## 5 Mechanisms of TANs involved in the development of breast cancer

### 5.1 Promotion of breast cancer by TANS

**Figure 2:**
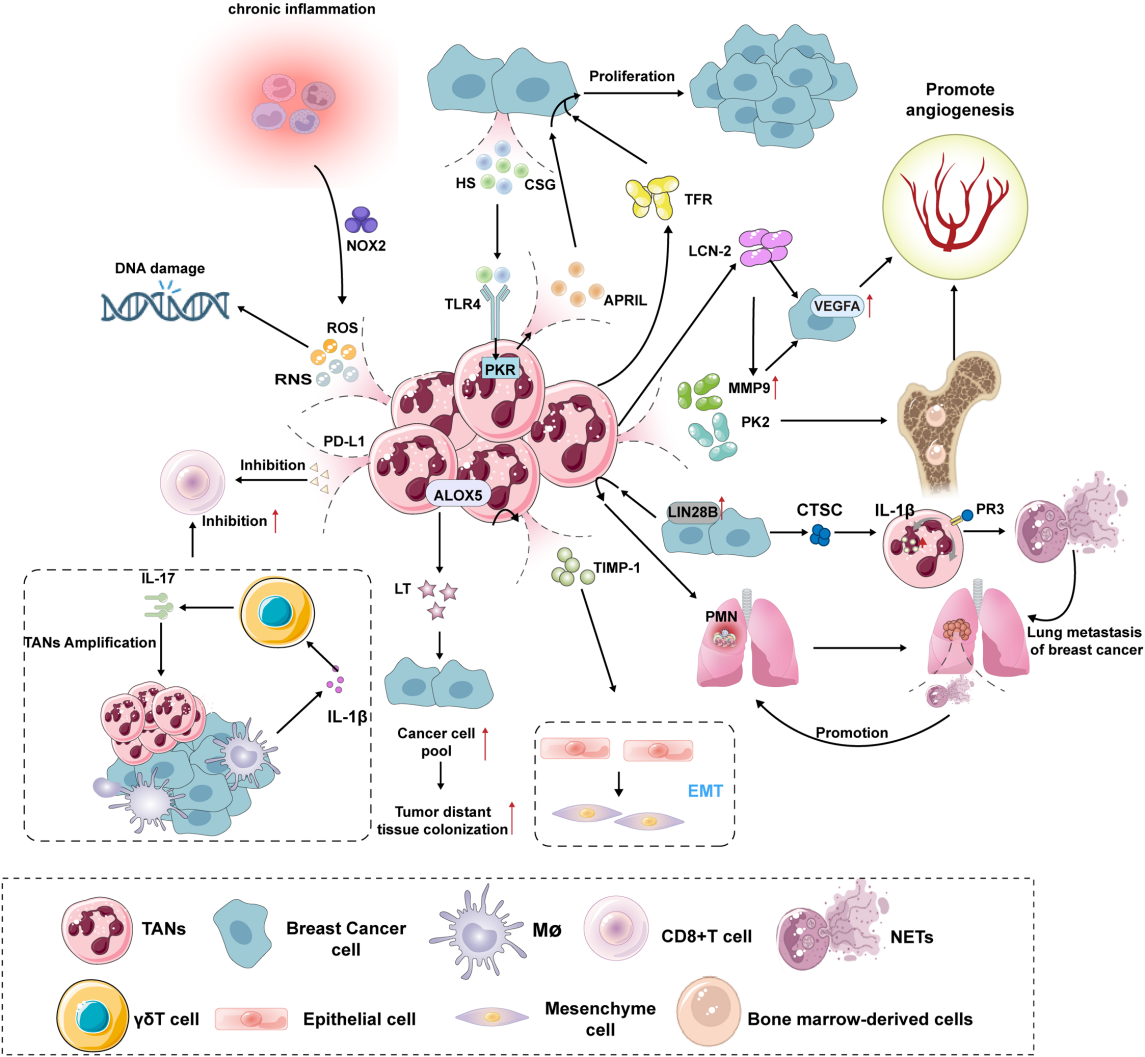
Mechanisms of TANs promoting the growth, proliferation and metastasis of breast cancer. TANs promotes the growth and proliferation of breast cancer: The release of ROS and RNS by TANs can induce DNA damage in infiltrating cells within the TME, leading to a significant increase in genomic instability and thereby elevating the risk of breast cancer development. When the body is in a chronic inflammatory state, it activates NOX2 in neutrophils, further amplifying the production of ROS/RNS, thus forming a cancer-promoting vicious cycle. HS and CSG induce PKR involvement via the TLR4 signaling pathway, promoting the secretion of APRIL by TANs, which stimulates the proliferation and growth of human breast cancer cells. Additionally, TANs can enhance breast cancer cell proliferation through the secretion of TFR-related factors. TANs promotes angiogenesis in breast cancer: TANs degrade the extracellular matrix by secreting MMP-9, thereby releasing and activating VEGF. Concurrently, TANs produce the pro-angiogenic factor PK2 (Prokineticin-2/Bv8), promoting angiogenesis. The hypoxic tumor microenvironment activates HIF-1α, upregulating VEGF expression. Breast cancer cells further amplify the VEGF signaling pathway through the LCN-2-mediated ERK/HIF-1α pathway. TANs promotes metastasis of breast cancer: High expression of LIN28B in breast cancer patients’ tumors recruits TANs to the lungs, establishing an immunosuppressive PMN that promotes breast cancer metastasis to the lungs. TANs secrete TIMP-1 to promote EMT in cancer cells, while EMT cells reciprocally enhance TAN activity through the Thy-1 signaling pathway, forming a pro-metastatic vicious cycle. Additionally, ALOX5 metabolites, LTs, selectively expand cancer cell populations with high tumorigenic potential, thereby improving the success rate of distant colonization. Anti-immune effect of TANs: TANs directly suppress the activity of CD8+ T cells and induce their functional exhaustion through the secretion of PD-L1. Additionally, TANs form a multicellular regulatory network with γδ T cells and macrophages, which drives the expansion of TANs via the IL-1β-IL-17 cascade and enhances their inhibitory effects on CD8+ T cells. Pro-tumor effects of NETs in breast cancer: NETs are highly enriched in breast cancer lung metastases, accelerating the awakening of dormant tumor cells and metastatic dissemination by promoting the formation of PMNs. Breast cancer cells secrete CTSC, which induces ROS and NET production in neutrophils through the CTSC-PR3-IL-1β axis, leading to THBS1 degradation, reduced endothelial cell adhesion, and ultimately promoting lung metastasis. ROS, Reactive Oxygen Species; RNS, reactive nitrogen species; NOX2, NADPH oxidase complex2; PD-L1, Programmed cell death 1 ligand 1; IL-17, Interleukin-17; IL-1β, Interleukin-1β; APRIL, A proliferation-inducing ligand; HS, Heparan Sulfate; CSG, Chondroitin Sulfate Glycosaminoglycan; TLR4, Toll-like receptor 4; PKR, RNA-activated protein kinase; ALOX5, Arachidonate 5-lipoxygenase; LT, Leukotriene; TIMP-1, tissue inhibitor of metal protease 1;TRF, Transferrin; VEGFA, vascular endothelial growth factor A; MMP9, matrix metalloproteinase 9; PK2, prokineticin 2; LCN-2, Lipocalin-2; PMN, pre-metastatic niche;EMT, Epithelial–mesenchymal transition; CTSC, tumorsecreted protease cathepsin C; PR3, Protease 3;NETs, Neutrophil extracellular traps.

TANs advance breast cancer progression by promoting tumor growth and metastasis, supporting tumor angiogenesis, immune suppression, and generating neutrophil extracellular traps (NETs) (**Figure 2**).

#### 5.1.1 TANs promote breast cancer development and cell proliferation

TANs promote breast carcinogenesis and cell proliferation through multiple mechanisms. The ROS and reactive nitrogen species (RNS) released by TANs can induce DNA damage in infiltrating cells within the TME, leading to significantly increased genomic instability, thereby elevating the risk of breast carcinogenesis (47). Notably, chronic inflammatory states can activate the neutrophil NADPH oxidase complex2 (NOX2), further amplifying the generation of reactive oxygen species/reactive nitrogen species (ROS/RNS), thereby forming a cancer-promoting vicious cycle (48). In the TME, TANs continuously drive tumor growth. Breast cancer cells exhibit high surface expression of heparan sulfate (HS) and chondroitin sulfate glycosaminoglycan (CSG), which act as endogenous ligands to specifically activate Toll-like receptor 4 (TLR4) on TANs. This activation triggers an RNA-activated protein kinase R (PKR)-dependent signaling cascade within neutrophils, promoting the secretion of a proliferation-inducing ligand (APRIL). APRIL binds to the transmembrane activator (TACI) on tumor cell surfaces, activating both the NF-κB and mitogen-activated protein kinase (MAPK) pathways, thereby driving abnormal proliferation of breast cancer cells (49, 50). Concurrently, transferrin (TFR) secreted by TANs binds to the transferrin receptor (TFRC) on breast cancer cells, activating iron-dependent signaling pathways. This process not only enhances the expression of cyclins (Cyclin D1/E) but also stimulates ribosomal biogenesis via activation of the mammalian target of rapamycin (mTOR) pathway, ultimately leading to a significant increase in tumor cell mitotic rates (51, 52).

#### 5.1.2 TANs promote angiogenesis in breast cancer

The angiogenic mechanisms in breast cancer involve a complex process coordinated by multiple factors through synergistic regulation. The vascular endothelial growth factor (VEGF) family, particularly VEGF-A, serves as a central regulatory factor. By binding to VEGFR-2 on endothelial cell surfaces, it activates downstream signaling pathways (e.g., PI3K/AKT, ERK), promoting endothelial cell proliferation, migration, increased vascular permeability, and ultimately driving angiogenesis (53). TANs enhance angiogenesis by secreting MMP-9, which degrades the extracellular matrix to release and activate VEGF. Simultaneously, TANs produce pro-angiogenic factors like prokineticin 2 (PK2; also known as BV8), which mobilize bone marrow-derived cells and stimulate endothelial cell division, significantly amplifying vascular formation (54, 55). The hypoxic tumor microenvironment activates hypoxia-inducible factor 1 alpha (HIF-1α), upregulating VEGF expression (56). Breast cancer cells further amplify VEGF signaling through the Lipocalin-2 (LCN-2)-mediated ERK/HIF-1α pathway (57). Vasculogenic mimicry (VM) formation relies on VEGFR-2/3-mediated upregulation of MMP9 (58). Additionally, receptor tyrosine kinases such as PDGFR-β and FGFR-2 synergize with VEGFR to promote vascular maturation and stabilization (59). Notably, in advanced stages, tumors evade single-target anti-VEGF therapies through compensatory mechanisms involving FGF-1, TGF-β, and other factors, underscoring the necessity for multi-target interventions.

#### 5.1.3 TANs promote breast cancer metastasis

The formation of the pre-metastatic niche (PMN) in breast cancer is a core mechanism for successful tumor cell colonization of distant organs, involving coordinated interactions among multiple molecular and cellular networks. Primary tumors induce vascular permeability in distant organs (via ZO-1 downregulation to disrupt endothelial barriers) and extracellular matrix (ECM) remodeling (e.g., fibronectin [FN] and tenascin-C [TnC] deposition) through the secretion of cytokines (e.g., VEGF, TGF-β) and exosomes (e.g., Cav-1-containing exosomes), thereby creating anchoring sites for metastatic cells (60, 61). Concurrently, tumor-derived factors (e.g., GM-CSF) regulate the nuclear translocation of the aryl hydrocarbon receptor (AHR) in macrophages, which binds to the PD-L1 promoter to upregulate its expression, driving regulatory T cell (Treg) differentiation and establishing an immunosuppressive PMN (62).Meyany et al. (63) reported that high expression of LIN28B in tumors of mice and breast cancer patients recruits tumor-associated neutrophils (TANs) to the lungs, establishes an immunosuppressive pre-metastatic niche (PMN), and promotes the spread of breast cancer to the lungs. TANs promote epithelial-mesenchymal transition (EMT) through tissue inhibitor of metal protease1 (TIMP-1) secretion, while EMT cells reciprocally enhance TAN activity via Thy-1 signaling, forming a pro-metastatic vicious cycle (64, 65). Additionally, the arachidonic acid 5-lipoxygenase (ALOX5) metabolite leukotriene (LT) selectively expands cancer cell populations with high tumorigenic potential, enhancing the success rate of distant colonization (66). PMN formation further involves hypoxia-mediated exacerbation of EMT and angiogenesis via HIF-1α, as well as CXCL12/CXCR4 chemotaxis pathways that mediate organ-specific homing (67). Combined interventions targeting critical nodes such as the AHR-PD-L1 axis, ALOX5 activity, and S100A6-mediated neutrophil-lymphatic endothelial cell interactions hold promise as novel therapeutic strategies to block PMN formation and suppress metastasis.

#### 5.1.4 Anti-immune effects of TANs

TANs mediate immune suppression and promote tumor progression in breast cancer through multiple mechanisms. First, TANs directly inhibit CD8+ T cell activity by secreting PD-L1 and induce their functional exhaustion. This process is regulated by cytokines such as C-C motif chemokine ligand 20 (CCL20) and interferon-γ (IFN-γ), and clinical studies confirm that PD-L1-positive tumors show significantly improved response rates to anti-PD-L1 antibody therapy (68). Second, TANs compete with T cells for microenvironmental resources through metabolic reprogramming. For example, the G-CSF-activated PI3K-AKT/NFκB signaling axis prolongs neutrophil lifespan and enhances their pro-metastatic capacity (69), while factors secreted by TANs, such as Interleukin-10 (IL-10) and arginase, directly suppress T cell proliferation (70). Additionally, TANs form a multicellular regulatory network with γδ T cells and macrophages. The IL-1β-IL-17 cascade drives TAN expansion and enhances their suppression of CD8+ T cells (69). Meanwhile, TANs further weaken T cell cytotoxicity through the JAG2-Notch signaling pathway or ROS release (71). Current therapeutic strategies include targeting PD-1/PD-L1 combined with chemotherapy or epigenetic modulators (e.g., CBP/P300 BRD inhibitors), which improve TME heterogeneity and reverse T cell exhaustion. Furthermore, CXCR2 inhibitors combined with immune checkpoint blockade (ICB) enhance efficacy by reprogramming TAN phenotypes (72).

#### 5.1.5 TANs induce drug resistance in breast cancer

TANs can induce chemotherapy resistance in breast cancer cells, leading to poor therapeutic outcomes after chemotherapy. Studies have revealed that chemotherapy-induced neutrophil extracellular traps (NETs) capture tumor-derived TGF-β via integrin αvβ1 and activate this factor using MMP-9, further promoting the EMT process, thereby reducing the chemotherapy efficacy against breast cancer lung metastasis (73). Additionally, neutrophil depletion experiments demonstrated that TANs directly enhance the EMT phenotype of tumor cells by secreting the chemokine CXCL-1 (74). During tumor progression, the EMT process significantly enhances tumor cell resistance to chemotherapeutic drugs by promoting the expression of EMT-related transcription factors such as E-box binding homeobox 1 (ZEB1), E-box binding homeobox 2 (ZEB2), and TWIST1 (75, 76). Intermediate-state EMT cells (hybrid epithelial-mesenchymal phenotype) exhibit greater plasticity, enabling them to evade chemotherapy-induced killing and rebuild metastatic niches through reversion to an epithelial state (77). Dynamic regulation of core transcription factors like Zeb1 and Snail/Slug is critical for driving bidirectional EMT/MET transitions (78). Furthermore, TANs in breast cancer patients undergoing chemotherapy display more pronounced senescence. These senescent TANs upregulate the expression of fat mass and obesity-associated protein (FTO) in breast cancer cells via secretion of exosomal piwi-interacting RNA-17560 (piR-17560).This process reduces RNA N6-methyladenosine (m6A) methylation levels, thereby promoting ZEB1 transcription factor expression and ultimately leading to chemoresistance (79, 80). Notably, ZEB1 expression is not only regulated by the FTO-m6A axis but also synergistically binds to the SNAIL1 promoter through the HMGA2/Smad complex, forming a positive feedback loop that amplifies EMT signaling (81). Future research should focus on targeting EMT plasticity, such as using histone deacetylase inhibitors (e.g., quisinostat) to downregulate ZEB1 or employing oxidative phosphorylation inhibitors to disrupt metabolic reprogramming in drug-resistant cells.

#### 5.1.6 Neutrophil extracellular traps

Neutrophils contribute to breast cancer progression through three primary mechanisms: first, by directly phagocytosing pathogens, which is the earliest recognized classical mechanism; second, by mediating cytotoxic effects through the release of toxic enzyme granules; third, by forming NETs—a specialized extracellular composite structure composed of cytoplasmic proteins, granule proteins, and chromatin (82, 83). Studies demonstrate that breast cancer cells and their TME are more prone to inducing NETs release compared to normal tissues, a phenomenon confirmed in both mouse models and breast cancer patients (84–87). NETs influence breast cancer progression via multiple molecular mechanisms. Active neutrophil elastase (NE) and MMP9 in NETs hydrolytically remodel LN, exposing specific epitopes that bind to dormant tumor cells, awakening them and initiating proliferation (88). Park et al (89). further confirmed that NETs are highly enriched in breast cancer lung metastases, accelerating the activation and metastatic spread of dormant tumor cells by promoting the formation of the PMN, particularly in TNBC patients. Additionally, breast cancer cell-secreted cathepsin C (CTSC) induces neutrophil production of ROS and NETs via the CTSC-PR3-IL-1β axis, leading to thrombospondin-1 (THBS1) degradation and reduced endothelial adhesion, ultimately facilitating lung metastasis (14). Notably, NETs are also highly enriched during breast cancer liver metastasis. Their DNA components bind to coiled-coil domain-containing protein 25 (CCDC25), activating the ILK-β-parvin signaling pathway, enhancing tumor cell invasiveness, and driving liver metastasis. Clinical data reveal that high CCDC25 expression in breast cancer patients correlates with shorter survival, identifying CCDC25 as a potential therapeutic target. Experimental studies confirm that targeted inhibition of CCDC25 significantly reduces NET-mediated distant organ metastasis, providing a theoretical basis for novel anti-metastatic strategies (90). In summary, NETs promote breast cancer metastasis through mechanisms such as matrix remodeling, signaling pathway activation, and microenvironment modulation. Targeted interventions against key NET effectors (e.g., NE, MMP9) and receptors (e.g., CCDC25) may emerge as novel therapeutic avenues to suppress metastasis.

### 5.2 Inhibitory effect of TANS on breast cancer

However, TANs still exhibit positive aspects. Firstly, TANs trigger anti-tumor immune responses by inducing tumor cell shedding, releasing nitric oxide (NO), and Fc-mediated plasma membrane uptake, thereby exerting anti-tumor activity (91). TANs can also recruit and activate immune cells by producing various mediators (such as cytokines, chemokines) and enzymes, stimulating T-cell proliferation, promoting NK cell and dendritic cell maturation, and resisting breast cancer cell invasion through the construction of an anti-tumor microenvironment (27, 92, 93). In a cyclin E-overexpressing breast cancer model, Elizabeth et al. discovered that NE released by neutrophils internalized by breast cancer cells hydrolyzes cyclin E to generate an HLA-A2-restricted peptide (ILLDWLMEV), which is recognized by cytotoxic T lymphocytes (CTLs) to kill breast cancer cells (94). Current research on the anti-cancer role of TANs in TNBC is limited. However, Frontera et al (69). reported that IgA Fc-folate conjugates, by binding to FR-α, induce ADCC in PMN-MDSCs, effectively killing TNBC cells. Since FR-α is minimally or not expressed in normal tissues, targeting FR-α reduces toxicity, providing a theoretical basis for FR-α as an ideal therapeutic target in TNBC (95). Finally, although substantial evidence suggests that neutrophils promote tumor metastasis by establishing an immunosuppressive microenvironment, anti-tumor TANs prevent metastasis by producing cytotoxic substances. In breast cancer, large numbers of TANs accumulate in the lungs before metastatic cells arrive, inhibiting the formation of metastatic niches by generating hydrogen peroxide to block tumor cell colonization (96). The study of the mechanisms of TANs in breast cancer exhibits significant dynamic complexity. Previous research predominantly focused on their pro-tumorigenic functions, yet recent advancements in single-cell sequencing technologies have unveiled the heterogeneity of neutrophils, gradually elucidating their anti-tumor mechanisms. Furthermore, the anti-tumor functions of neutrophils may manifest prominently only under specific conditions, such as early-stage tumors or particular immunotherapeutic strategies. In contrast, TANs in advanced tumors are often “educated” by the TME to adopt a pro-tumorigenic phenotype. For instance, in breast cancer TME, the T3 neutrophil subset enriched in hypoxic core regions exhibits glycolytic features strongly correlated with pro-angiogenic activity, while anti-tumor subsets predominantly localize to the tumor periphery (97).

## 6 Targeted therapeutic strategies against TANs in breast cancer

### 6.1 Regulating pro-tumor TANs

The phenotypic polarization and functional regulation of TANs hold significant importance in cancer therapy. TGF-β is a key driver of TANs polarization toward a pro-tumor phenotype. Its signaling pathway promotes tumor progression by inducing EMT and chemotherapy resistance (73). Inhibiting TGF-β signaling can reverse pro-tumor TANs polarization, restore neutrophil cytotoxicity, and enhance anti-tumor immune responses. On the other hand, type I interferons (IFN-α/β) significantly promote TANs polarization toward an anti-tumor phenotype by activating the Fas/FasL pathway, inducing apoptosis-related genes in pro-tumor TANs, and upregulating effector molecules such as NO, H_2_O_2_, and TNF (98, 99). NETs play a critical role in tumor metastasis. Targeting NET-related enzymes (e.g., peptidylarginine deiminase 4/PAD4) or degrading free DNA via DNase can effectively inhibit the pro-metastatic effects of NETs (100). Interventions targeting TANs metabolic reprogramming also show therapeutic potential. For example, inhibiting fatty acid transport protein 2 (FATP2) reduces the synthesis of immunosuppressive lipid mediators, thereby reversing the pro-tumor phenotype and delaying breast cancer progression (101). In combination therapies, enhancing ferroptosis-related pathways may boost TANs anti-tumor activity by increasing ROS levels, though the specific mechanisms require further validation (102). In immune checkpoint inhibitor-based therapies, CD47-SIRPα blockers enhance tumor cell clearance through TANs-mediated trogocytosis of antibody-labeled tumor cells, while targeting FcγRIIa (CD32a) and FcαRI (CD89) optimizes antibody-dependent cellular cytotoxicity (ADCC) (103). Additionally, combining PD-1/PD-L1 inhibitors with TANs-targeted therapies (e.g., Chi3l1 inhibitors) significantly improves immunotherapy efficacy in triple-negative breast cancer by counteracting TANs-mediated immune exclusion (104).

### 6.2 Antibody therapy

Monoclonal antibodies (such as rituximab and trastuzumab) bind to Fcγ receptors (e.g., FcγRIIa) on the surface of neutrophils via their Fc regions, triggering ADCC to directly kill tumor cells. Studies (105) have shown that polymorphisms in FcγRIIa significantly affect therapeutic efficacy, with breast cancer patients carrying the high-affinity H131 genotype (H/H) exhibiting better clinical responses to trastuzumab. Additionally, TANs can progressively phagocytose tumor cell membranes through “trogocytosis,” leading to mechanical disruption of tumor cells (106). When combined with CD47 inhibitors (e.g., anti-CD47 monoclonal antibodies), the CD47-SIRPα signaling axis on tumor cells is blocked, lifting the inhibitory signals on TANs and significantly enhancing their phagocytic efficiency (107). Preclinical studies demonstrate that IgG1 antibodies combined with CD47 inhibitors improve the tumor-killing efficacy of TANs against breast cancer cells. However, IgA antibodies show superior potential due to their high-affinity binding to FcαRI and the absence of inhibitory receptor interference (106). Specifically, CD47-SIRPα blockade increases IgA-mediated neutrophil cytotoxicity to 80%, compared to only 40% with IgG1 alone. Notably, high CD47 expression correlates with poor responses to trastuzumab in breast cancer patients, likely due to tumor immune evasion via the CD47-SIRPα axis. Blocking CD47 reverses this resistance, as demonstrated in HER2-positive breast cancer models where trastuzumab efficacy was restored (108). Furthermore, this combination therapy not only enhances macrophage-mediated antibody-dependent cellular phagocytosis (ADCP) but also promotes neutrophil-mediated ADCC. It also improves overall antitumor responses by modulating immune checkpoints in the tumor microenvironment (109). Currently, combination therapies targeting the CD47-SIRPα axis have demonstrated synergistic antitumor effects in multiple clinical trials.

### 6.3 The therapeutic potential of engineered TANs

Neutrophils, leveraging their unique inflammatory chemotaxis and barrier-crossing abilities, can load chemotherapeutic drugs or nanoparticles, demonstrating significant advantages in tumor-targeted therapy. When combined with photothermal therapy (PTT), near-infrared (NIR) light irradiation not only induces localized drug release but also recruits more neutrophils to the tumor site through acute inflammation triggered by photothermal effects, creating synergistic antitumor effects (110). Studies show that neutrophil infiltration at the tumor site increases by 2.8-fold post-PTT, accompanied by significantly elevated pro-inflammatory cytokine levels, further enhancing drug-targeted delivery efficiency (111). Additionally, neutrophil membrane-coated nanoparticles (NM-NPs) inherit the CD47 molecules and membrane protein characteristics of parent cells, extending circulation time by over 5-fold and improving tumor accumulation by mimicking neutrophil chemotaxis (110). The application of gene-editing technologies further expands the therapeutic potential of neutrophils. CRISPR/Cas9-mediated knockout of the immunosuppressive gene Arg1 alleviates TME-imposed functional restrictions on neutrophils, while overexpression of pro-apoptotic factors like TRAIL directly enhances their cancer-killing capacity (110). Experimental data indicate that adoptively transferred gene-edited neutrophils increase T-cell infiltration in tumors by 3-fold, and their combination with PD-1 inhibitors significantly prolongs survival in murine models. These strategies integrate neutrophils’ innate biological properties with engineered modifications, offering novel approaches to overcome limitations of conventional therapies in solid tumor treatment.

## 7 TANs and complications in breast cancer patients

Venous thromboembolism (VTE) is the second leading cause of death in cancer patients (112). Cancer patients often exhibit a hypercoagulable state, even in the absence of overt thrombosis. Citrullinated histone H3 (Cit H3), as a biomarker of NETs formation, has higher plasma levels, circulating free DNA, myeloperoxidase, and Cit H3 in mice with breast tumors compared to tumor-free mice. Cit H3 is associated with VTE in cancer patients (113, 114). NETs can mediate the formation of a prothrombotic state in tumor patients through various mechanisms, ultimately leading to thrombus formation. As a macromolecular complex that forms a mesh-like structure, NETs provide a scaffold for red blood cells and various procoagulant factors, thereby promoting thrombin generation. NETs can also capture platelets and promote platelet activation, adhesion, and aggregation, increasing blood viscosity and leading to thrombus formation (115). TANs have rarely been reported to cause damage to primary breast cancer sites (rare metastatic sites) and areas not directly affected by tumors. However, using a mouse model of murine mammary tumor virus-polymyeloma intermediate tumor antigen, Cedervall et al. found that neutrophil infiltration in non-tumor common metastatic sites led to significant vascular lesions within organs, with a notable increase in TANs in the hearts and kidneys of mice, and significant impairment of vascular function in these organs; when TANs were cleared, vascular function returned to normal (116).

## 8 Prognosis of TANs

The neutrophil-to-lymphocyte ratio (NLR) has emerged as a novel biomarker for disease assessment and has become a prominent area of biomedical research. While the NLR is widely recognized as an indicator of immune system homeostasis, precise and independent cut-off values have not yet been established. Studies demonstrate that elevated NLR serves as a marker of poor prognosis in both TNBC and HER2-positive breast cancer, with increasing ratios correlating with heightened mortality risk in breast cancer patients (117). Dynamic changes in NLR show close associations with time to recurrence and mortality in TNBC patients (118–120). Consequently, implementing dynamic clinical monitoring of NLR, timely tracking of prognostic outcomes, and optimizing therapeutic regimens may further extend patient survival. In recent years, some researchers have proposed through follow-up studies and model construction that levels of NETs in peripheral blood could also serve as prognostic indicators in breast cancer (121–123). Elevated levels of NET-derived DNA in early-stage breast cancer patients correlate with subsequent hepatic metastasis, while patient responses to chemotherapeutic agents and immunotherapies are closely associated with the expression of NET-related long non-coding RNAs (lncRNAs). These findings underscore the significant clinical value of NETs, warranting in-depth investigation.

In recent years, many scholars have adopted the view that NETs are the main reason why neutrophils play a dual role in the tumor microenvironment. Accordingly, this review has summarized the role and significance of these NETs in the occurrence, development, and prognosis of breast cancer (**Table 1**).

**Table 1:** Role of NETs in the development and prognosis of breast cancer.

Action mechanism	Site of action	Mode of action	Ref.
Augmentation of breast cancer	Lung	Hydrolyzed LN, exposed associated epitopes and bound to dormant tumor cells.	(79)
Facilitation of transfer	Lung	Affect the growth of PMN, promote the awakening of dormant cancer cells.	(12)
		Degrades THBS1 and reduces endothelial cell adhesion.	(80)
	Liver	NET DNA binds to CCDC25 to activate the ILK-β-parvin pathway and enhance the aggressive behavior of cancer cells.	(80)
promotion of thrombosis	Blood vessel	Provide scaffolds for red blood cells and pro-coagulant factors to promote thrombin formation.	(92)
		Capture platelets, and promote platelet activation, adhesion, and aggregation.	
Organism prognosis	Blood vessel	High levels of NET DNA in the blood are associated with liver metastasis.	(97, 98)
		The response of patients to chemotherapy drugs and immunotherapy was closely related to the expression of lncRNAs associated with NETs.	(99)

## 9 Discussion

Although targeting TANs provides novel insights for precision intervention in breast cancer, their clinical translation still faces multiple challenges. First, the high heterogeneity of TANs leads to significant phenotypic and functional diversity among their subpopulations, making single-target strategies insufficient to comprehensively regulate their pro-tumor or anti-tumor effects. This necessitates the development of combination therapies based on multi-pathway synergistic inhibition, such as CXCR2 inhibitors combined with epigenetic regulators (e.g., HDAC inhibitors). Second, existing prognostic markers like the NLR in peripheral blood, while associated with breast cancer survival rates, exhibit significantly variable predictive efficacy across molecular subtypes. To address this, it is critical to integrate single-cell transcriptomic data with clinicopathological features to construct subtype-specific predictive models, particularly optimizing risk stratification in aggressive subtypes such as TNBC. Furthermore, the dynamic functional switching of TANs is closely linked to disease progression stages: early-stage TANs may suppress tumor immune surveillance via ROS release, whereas late-stage TANs tend to promote pre-metastatic niche formation. This underscores the need to precisely determine intervention timing by combining radiomics and liquid biopsy technologies, such as monitoring tumor burden via circulating tumor DNA (ctDNA) to guide a “suppress early, activate late” time-sequential therapeutic strategy. To resolve these issues, future research should integrate single-cell multi-omics with spatial transcriptomics to deeply resolve the heterogeneity of TANs and their dynamic roles in the TME. Specifically, single-cell sequencing can identify surface markers specific to TANs subpopulations and reveal their phenotypic switching networks across different breast cancer stages, including the coexistence states of pro-tumor and anti-tumor subpopulations, stage-specific dominant modes, and imbalance-triggering mechanisms. Meanwhile, spatial transcriptomics can compensate for the spatial information loss in single-cell sequencing by mapping interaction sites between TANs and tumor, immune cells (e.g., macrophages and T cells), thereby deciphering spatial distribution features of signaling microenvironments. This multi-dimensional integration will not only elucidate the spatiotemporal dynamics of TANs but also uncover their collaborative networks with stromal cells and immunosuppressive myeloid cells. Ultimately, these findings will provide theoretical foundations for developing spatiotemporally targeted therapies and optimizing immunocombination strategies to achieve precise regulation from “tumor promotion suppression” to “anti-tumor activation”.

## Glossary

TANs: tumor-associated neutrophils
TME: tumor microenvironment
CXCR2: CXC receptor 2
G-CSF: Granulocyte Colony Stimulating Factor
NFAT1: Nuclear Factor of Activated T-cells 1
CXCL8: CXC Motif Chemokine Ligand-8
CTSC: tumor-secreted protease cathepsin C
PR3: Protease 3
IL-1β: Interleukin-1β
NF-κB: nuclear factor-kB
IL-6: Interleukin-6
CCL3: CC Motif Chemokine Ligand-3
IL-17: Interleukin-17
FR-α: Folate Receptor Alpha
TNBC: triple negative breast cancer
GRO: growth-regulated oncogene
CXCL1: CXC Motif Chemokine Ligand-1
CXCL2: CXC Motif Chemokine Ligand-2
CXCL3: CXC Motif Chemokine Ligand-3
TGF-β: Transforming Growth Factor Beta
TNF-α: Tumour Necrosis Factor Alpha
MSCs: mesenchymal stromal cells
CXCL5: CXC Motif Chemokine Ligand-5
CXCL8: CXC Motif Chemokine Ligand-8
IFN-α: interferon-α
IFN-β: interferon-β
IFN-γ: interferon-γ
ROS: Reactive Oxygen Species
NO: Nitric Oxide
ADCC: antibody-dependent cellular cytotoxicity
scRNA-seq: single-cell RNA sequencing
ATAC-seq: assay for transposase-accessible chromatin sequencing
IORT: intraoperative radiotherapy
HR: hormone receptor
LTB4: Leukotriene B4
ARG1: Arginase 1
LDNs: low-density neutrophils
HDNs: high-density neutrophils
iLDNs: immature low-density neutrophils
MDSCs: myeloid-derived suppressor cells
G-MDSCs: Granulocytic MDSCs
MMPs: matrix metalloproteinases
TAMs: tumor-associated macrophages
PMN-MDSCs: polymorphonuclear-MDSCs
RNS: reactive nitrogen species
iNOS: inducible nitric oxide synthase
NOX2: NADPH oxidase complex2
APRIL: A proliferation-inducing ligand
MAPK: mitogen-activated protein kinase
HS: Heparan Sulfate
CSG: Chondroitin Sulfate Glycosaminoglycan
TLR4: Toll-like receptor 4
PKR: RNA-activated protein kinase
TRF: Transferrin
TFRC: transferrin receptor
VEGFA: vascular endothelial growth factor A
MMP9: matrix metalloproteinase 9
PK2: prokineticin 2
LCN-2: Lipocalin-2
HIF-1α: hypoxia-inducible factor 1 alpha
VM: Vasculogenic mimicry
Erk: extracellular signal-regulated kinase
ECM: extracellular matrix
ECM: extracellular matrix
VEGF: vascular endothelial growth factor
PMN: pre-metastatic niche
E-Cad: E-Cadherin
FN: fibronectin
TnC: tenascin-C
EMT: Epithelial–mesenchymal transition
AHR: aryl hydrocarbon receptor
TIMP-1: tissue inhibitor of metal protease 1
ALOX5: Arachidonate 5-lipoxygenase
LT: Leukotriene
PKM2: pyruvate kinase M2
ICB: immune checkpoint blockade
PD-1: Programmed Death Receptor1
PD-L1: Programmed cell death 1 ligand 1
CCL20: C-C motif chemokine ligand 20
IL-10: Interleukin-10
ZEB1: E-box binding homeobox 1
ZEB2: E-box binding homeobox 2
FTO: obesity-associated protein
m6A: RNA N6-methyladenosine
CTLs: cytotoxic T lymphocytes
NETs: Neutrophil extracellular traps
LN: laminin
NE: neutrophil elastase
THBS1: thrombospondin-1
VTE: Venous thromboembolism
Cit H3: citrullinated histone H3
NLR: neutrophil-to-lymphocyte ratio
lncRNAs: long non-coding RNAs
ctDNA: circulating tumor DNA
FATP2: fatty acid transport protein 2
ADCP: antibody-dependent cellular phagocytosis
PTT: photothermal therapy.
